# Iron Oxide Impregnated *Morus alba* L. Fruit Peel for Biosorption of Co(II): Biosorption Properties and Mechanism

**DOI:** 10.1155/2013/917146

**Published:** 2013-11-10

**Authors:** Janardhan Reddy Koduru, Yoon-Young Chang, Jae-Kyu Yang, Im-Soon Kim

**Affiliations:** ^1^Graduate School of Environmental Studies, Kwangwoon University, Wolgye-Dong, Nowon-Gu, Seoul 139-701, Republic of Korea; ^2^Department of Environmental Engineering, Kwangwoon University, Seoul 139-701, Republic of Korea; ^3^Division of General Education, Kwangwoon University, Seoul 139-701, Republic of Korea

## Abstract

Biosorption is an ecofriendly wastewater treatment technique with high efficiency and low operating cost involving simple process for the removal of heavy metal ions from aqueous solution. In the present investigation, *Morus alba* L. fruit peel powder (MAFP) and iron oxide impregnated *Morus alba* L. fruit peel powder (IO-MAFP) were prepared and used for treating Co(II) contaminated aqueous solutions. Further the materials were characterized by using FTIR and SEM-EDX analysis. From FT-IR analysis it was found that hydroxyl, methoxy, and carbonyl groups are responsible for Co(II) biosorption. The kinetic data obtained for both biosorbents was well fitted with pseudo-second-order kinetic model. The equilibrium data was in tune with the Langmuir and Freundlich isotherm models. The thermodynamic studies were also carried and it was observed that sorption process was endothermic at 298–328 K. These studies demonstrated that both biosorbents were promising, efficient, economic, and biodegradable sorbents.

## 1. Introduction

In recent years the presence of metal ions in aquatic environment is of utmost importance, due to their almost indefinite persistence in nature and most of them are being toxic. Among various toxic metal ions cobalt is one of the hazardous element that affects the environment [1]. Cobalt enters into the aquatic environment through several industrial activities such as nuclear power plants, metal plating, and mining and fertilizer industry [2, 3]. The presence of cobalt in higher concentration may cause several serious health problems such as paralysis, diarrhea, low blood pressure, lung irritation, and bone defects [1, 4]. It may also cause damage to the thyroid and liver and also genetic changes in living cells [5–9]. Due to its toxicity various regulatory bodies have set permissible limits for cobalt in drinking water. The tolerance limits of Co(II) in potable/irrigation water and livestock waste water have been fixed as 0.05 mg L^−1^ and 1.0 mg L^−1^ [1, 10–14].

Therefore, it is necessary to treat the Co(II) present in industries waste water before being discharged into natural water. Several methods have been developed and employed for treating Co(II) from aqueous solutions [12–14]. However most of these conventional methods are restricted in some aspects, such as disadvantages like incomplete metal ion removal, high reagent and energy consumption, generation of toxic sludge, or other waste products that require careful disposal. Hence, it imperative to develop a cost-effective treatment method that is capable of removing Co(II) from aqueous effluents [14–17]. In recent year biosorption technology has become one of the alternative methods that have been widely employed for treating various metal ions from aqueous solutions [18, 19]. In view of this, a number of researchers have used biosorption technology for the removal of Co(II) from aqueous environment [14, 15, 20–28]. Biosorption has potential marketing advantages over other traditional wastewater treatment technologies including low cost, high efficiency, minimization of chemical and low biological sludge, no additional nutrient requirement, regeneration of biosorbent along strong ability to bind metal ions, and possibility of metal recovery and environmental friendly, particularly when natural biomass is used [29]. Among the various biomaterials employed, natural biomass obtained from various fruits peels was used as potential biosorbent for removal of various pollutants, especially metal ions [30–33].

*Morus alba* L., known as white mulberry, is a short-lived, fast-growing, and small-to-medium sized mulberry tree, is native to northern China, and is widely cultivated and naturalized in China [34]. It belongs to *Moraceae* family and *morus* genus of *plantae* kingdom. Its species' extracts are widely used to treat prematurely grey hair, to “tonify” the blood and are also used to treat constipation, diabetes, cough, wheezing, and edema and promote urination, fever, headache, and red dry and sore eyes [34]. It is also used as antibacterial agent against food poisoning microorganism [35], as an indicator in acid-base titration [36] and also used to restore the vascular reactivity of diabetic patients and antiadherence activity [37]. The species are planted and grew throughout the world, such as India, Afghanistan, Iran, and southern European countries for over thousand years. It is mainly used as food for the silkworms and they are sometimes eaten as vegetable or used as cattle fodder in different parts of the world [34]. Along with its wide availability* Morus alba* L. also contains various chelating functional groups such as –OH, –COOH, amine amide, and –SH groups [38]. Recently, it has been used as biosorbent for the removal of Cd(II) at trace level [39]. Due to its wide availability, low cost, and presence of various chelating groups, *Morus alba* L. can be used as effective biomaterial for treating metal contaminated aqueous solutions. However, some of the researchers widely use metal oxides combined with various natural biomaterials for metal ion removal [40–42], whereas they can improve permeability and facilitate phase separation in flow-through systems [43]. It was observed that, metal oxide loaded materials were used as an alternative materials for effective sequestration of target metal ions [44, 45]. The aforesaid reasons prompted us to synthesize iron oxide impregnated *Morus alba* L. fuite peel (IO-MAFP) powder for the removal Co(II) from aqueous solution.

The aim of the present work was to examine the efficiency of *Morus alba *L. fruit peel powder (MAFP) and its iron oxide impregnated (IO-MAFP) compound for the removal of Co(II) from aqueous solutions. Various experimental parameters such as equilibrium, pH effect, biosorbent dosage, contact time, and initial concentration on Co(II) removal were studied. Equilibrium isotherm models like Langmuir, Freundlich, and Temkin were used to determine the mechanism of the biosorption process. The developed method was successfully applied for the removal of Co(II) from aqueous solutions. The present biosorbents are efficient and sensitive for the quantitatively sorption of Co(II) when compared to the reported biosorbents. 

## 2. Materials and Methods

### 2.1. Preparation of the Biosorbents

*Morus alba *L. fruit peel was obtained from oriental medical college, Gyeongju. The collected biomass was dried in open air and ground in a mill to get fine powder. The peel powder was washed twice with deionizer water and dried at 60°C for 24 hrs then boiled in double distilled deionizer water by changing the water repeatedly until water becomes colorless, which indicates the complete removal of water soluble color compounds. To make it mineral free, It was boiled with 2.0 moL L^−1^ hydrochloric acid on hot plate at 60°C then washed with double distilled deionizer water and dried at 60°C for 24 hrs. The washed fruit fine powder was dried in vacuum oven at 60°C for 24 hrs, was noted as MAFP, and was stored in desiccators to be prevented from moisture before it was used for biosorption of Co(II). 

**Figure 1:**
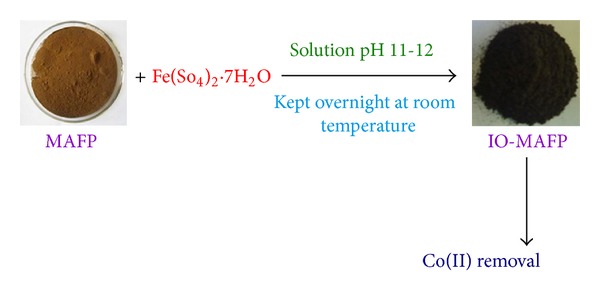
Schematic representation of preparation of iron oxide impregnated *Morus alb *L. fruit peel powder (IO-MAFP) sorbent.

Iron oxide impregnated* Morus alba* L. fruit peel powder was prepared as the following procedure (Figure 1). 1.0 moL L^−1^ FeSO_4_ *·*7H_2_O was added to the above washed *Morus alba* L. fruit peel powder and stirred for 30 min in a polyethylene vessel. To this, 2.0 M NaOH was added drop wise to get a pH of about 11.0–12.0 and then stored for 20 hrs at room temperature. Collected and washed, the formed iron oxide impregnated *Morus alba* L. fruit peel with 500 mL of distilled water was dried at room temperature and was named IO-MAFP.

### 2.2. Chemicals and Instruments

Analytical grade cobalt acetate (Co(CH_3_COO)_2_·4H_2_O) purchased from Sigma-Aldrich (Ireland) was used for the preparing 1000 mg L^−1^ Co(II) standard metal solution. It was diluted with distilled water for the required working experimental solution. pH 340i, WTW (Germany), was used for measuring solution pH. The concentration of metal ion was determined using Varian Spectra AA220 model atomic absorption spectrometer (AAS). All measurements were carried out in an air/acetylene flame. Energy dispersive X-ray spectroscopy coupled with scanning electron microscope (S-4300 & EDX-350, Hitachi, Japan) was used for the determination of morphology and composition of the biosorbents under following conditions: an SE resolution of 1.5 nm (at 15 kV) and 5.0 nm (at 1 kV) a magnification range of 20 to 500,000 at 0.5–30 kV accelerating voltage of an electron gun with a cold type field emission specimen stage dimension of *X*-*Y* (25 mm-25 mm), *Z* (5.0 mm to 30 mm), tilt (−5 to +45°), and rotation (360°) and equipped with EDX composition analyzer. Surface area and pore sizes of biosorbent were measured by Brunauer-Emmett-Teller method (BET) (Autosorb-1, Quanta chrome instrument, USA) based on N_2_ gas adsorption on the surface of biosorbent at 60°C. The FT-IR spectra of samples were examined using spectrum GX & Auto image (Perkin-Elmer, USA) at 4000 to 400 cm^−1^ spectral range using Ge coated KBr beam splitter with 0.25 resolution DTGS detector. The crystalline structure of iron oxide on each adsorbent was confirmed by XRD analysis containing a Cu K*α* (1.54059 Ǻ) source (40 kV, 100 mA; Siemens D-501 tube) using Rigaku D/Max-2500 X-ray diffractometer (Rigaku, Japan). Scanning was done in the 2*θ* range of 10–80 degrees at a scan speed of 6 degree min^−1^. 

### 2.3. Batch Biosorption Studies

0.05 g of biomass, MAFP, and IO-MAFP in 25 mL of the Co(II) metal ion solution at pH 6.0 in different conical flask was used for the batch biosorption studies. The batch biosorption studies were carried out in a temperature controlled shaker (Vision Scientific Co. Ltd., Republic of Korea) at 200 rpm and 25 ± 2°C. At the end of predetermined time interval the reaction mixtures were filtered out. The filtrate was analyzed using AAS for the determination of adsorbed metal ion concentration. The batch biosorption experiments were also conducted to determine the equilibrium time (10–60 min), initial concentrations (2.0–20 mg L^−1^), and adsorbent dosages (2.0–8.0 g L^−1^) to obtain the maximum adsorption capacity. All the investigations were carried out in duplicate to avoid any discrepancy in experimental results. To maintain quality control, the metal solution controls were used throughout the experiment. The metal sorption percentage of the biosorbents was measured using the following equation:

(1) $$\begin{array}{c} \text{Biosorption}\;\left(\%\right)=\left(\frac{C_{i}-\;C_{f}}{C_{i}}\right)\;\times 100, \end{array}$$

where *C*_*i*_, *C*_*e*_, and *C*_*f*_ are the initial, equilibrium, and final concentration of metal ion (mg L^−1^) in the solution, respectively. The amount of sorption capacity was calculated by using mass balance equation:

(2) $$\begin{array}{c} q_{e}=\frac{\left(C_{i}-\;C_{e}\right)\;V}{m}, \end{array}$$

where *q*_*e*_ is the adsorption capacity (mg g^−1^), *V* is the volume of metal ion solution (L), and *m* is the weight of the adsorbent (g).

### 2.4. Sorption Kinetic Studies

Kinetics studies were carried out in order to determine the sorption equilibrium time. 25 mL samples containing 2.0–20 mg L^−1^ Co(II) metal ion solutions at desired pH 6.0 was added to 2.0 g L^−1^ of biomass. Sorption processes were carried out in a flask placed in a thermostatic water bath shaker at 25 ± 2°C. The amount of sorption capacity at time *t* was measured using the following equation:

(3) $$\begin{array}{c} q_{t}=\frac{\left(C_{i}-\;C_{t}\right)\;V}{m}, \end{array}$$

where *C*_*t*_ (mg L^−1^) is the concentration of metal ion at particular time and *t* in the present experiment.

## 3. Results and Discussion

### 3.1. Characterization of Biosorbents

The bulk density, moisture content, ash content, surface area, surface composition, and other physical characteristics of the biomasses (MAFPand IO-MAFP) which influence biosorption of metal ion were measured and reported in Table 1. The bulk density of biomasses were found to be in the range 0.35~0.55 g mL^−1^ with 1.2~1.3% and 11.4~12.53% of the moisture and ash content, respectively, for both sorbents. The BET surface areas of biomass were found to be 16.29 and 335.24 m^2^ g^−1^ for MAFP and IO-MAFP, respectively, using N_2_ gas adsorption method. The increase in surface area of IO-MAFP may indicate the impregnation of iron oxide in to biosorbent, MAFP. The pore mean diameter (15.27 and 55.5 × 10^−8^ cm for MAFP and IO-MAFP resp.) of biomasses was confirmed as mesopores materials (20 × 10^−8^ cm < *d* < 500 × 10^−8^ cm, International Union of Pure and Applied Chemistry (IUPAC)).

**Figure 2:**
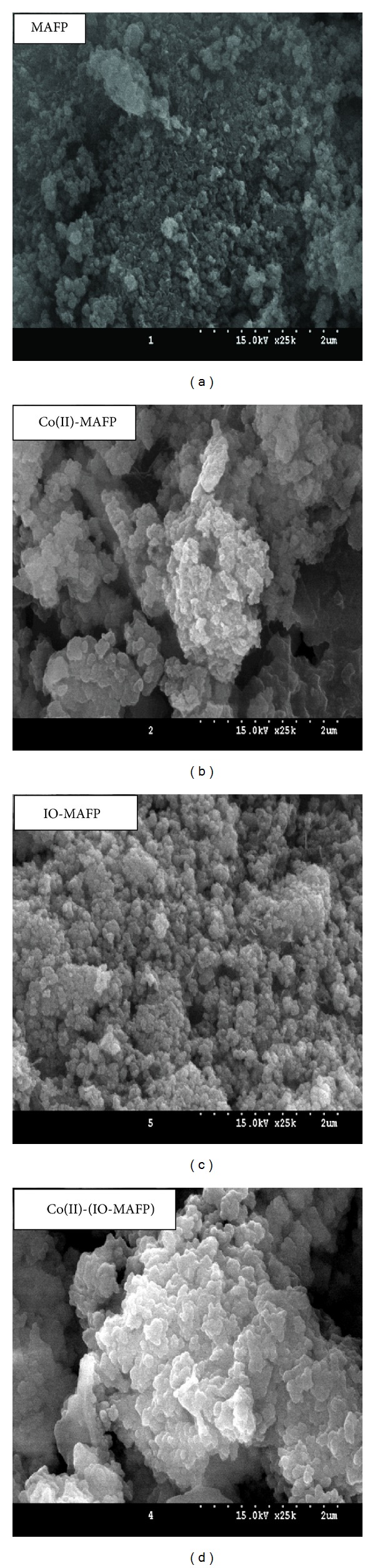
SEM images of MAFP and IO-MAFP before and after sorption Co(II).

**Figure 3:**
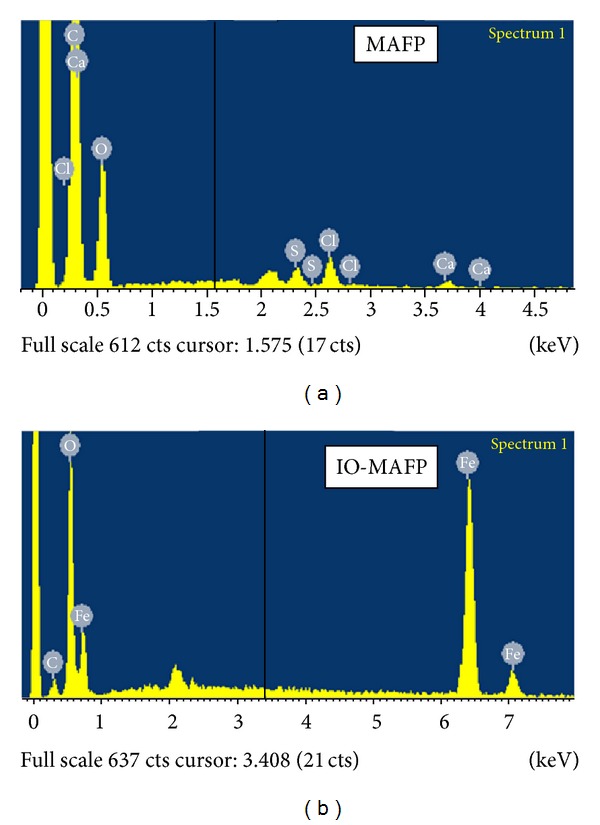
SEM-EDX spectrum of MAFP biomass and IO-MAFP.

The surface morphology of MAFP biomass and IO-MAFP biomass was shown in Figure 2. It's clear from SEM images that both biomasses have the rough surface morphology, which possesses possibility for more adsorption of Co(II) ions. The difference in the surface morphology of IO-MAFP from MAFP, slight white surface morphology, confirms the iron oxide impregnation onto MAFP. The surface morphology of both Co(II) loaded MAFP and IO-MAFP biomasses was more different than original biomasses (Figure 2). Shiny and bright white surface morphology indicates the Co(II) metal ions adsorbed on the surface of biomasses, MAFP, and IO-MAFP. The EDX spectrum (Figure 3) of MAFP biomass reveals that carbon (65.94 w%) and oxygen (29.45 w%) were relatively high surface elemental composition when compared to the calcium (0.73 w%), sulfur (1.35 w%), and chlorine (2.54 w%), whereas the IO-MAFP EDX spectrum reveals that the Fe (76.16 w%) and oxygen (20.42 w%) were the relatively high surface elemental composition when compared to carbon (3.43 w%) and other. This obtained results concluded that iron oxide was impregnated onto MAFP. Based on the above results, the biomasses, MAFP, and IO-MAFP are good and suitable for employing as biosorbent.

**Figure 4:**
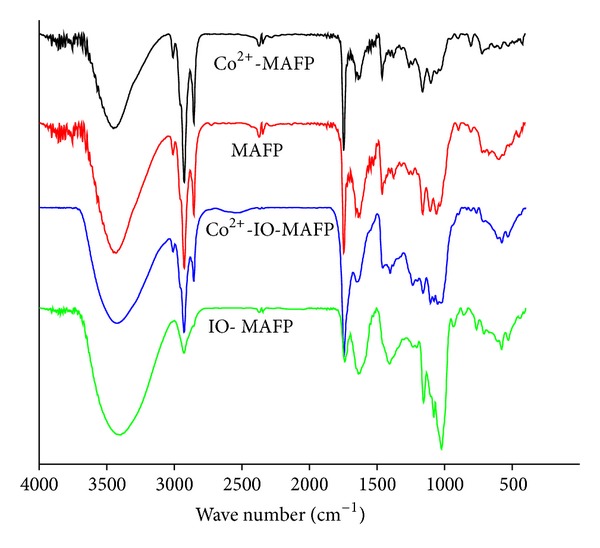
FT-IR spectrum of biomasses, MAFP, and IO-MAFP before and after Co(II) loaded.

The FT-IR spectrum of biomasses, MAFP, and IO-MAFP (Figure 4) exhibited a number of absorption peaks indicating the presence of various types of functional groups. In the FT-IR spectrum of MAFP, a broad and strong absorption peaks in the range 3407 to 3443 cm^−1^ are assigned to aromatic and aliphatic –NH_2_ and –OH groups. The peaks in the range 2990 to 3015 cm^−1^ indicate –C–H stretching vibrations in the hetero aromatic structure and the strong peaks at 2853.56 to 2943.44 cm^−1^ are related to the C–H vibration of alkyl and ethylene groups of side chains and aromatic methoxyl groups while the weak absorption peaks between 2343.22 and 2368.37 cm^−1^ indicate C–C triple bond at side chain of aromatic ring or aliphatic chains. The strong stretching absorption peaks in the range from 1743.04 to 1753.81 cm^−1^ are assigned as carbonyl stretching vibrations. The absorption peaks at 1638 to 1639 cm^−1^ indicate the aromatic ring alkenes and amide group –C–O stretching frequency while the peaks at 1466.31 to 1355.78 cm^−1^ are typical vibrations in alkynes skeleton on aromatic ring. The absorption peaks between 1272.27 and 1035.41 cm^−1^ indicate –C–O stretching vibrations in side chains of aromatic ring units and the vibrations bands at 927.24 to 704.40 cm^−1^ are indicating the presence of substituted phenyl rings. The absorption peaks observed between 650.49 and 420.49 cm^−1^ are related to C-X (X = Cl^−1^, S^2−^) stretching vibrations. After impregnation of iron oxide onto *Morus Alba* L. fruit peel (IO-MAFP), the FT-IR various absorptions peaks of MAFP, such as 3433.14, 2347.40, 1746.78, 1640.15, 1458.91, 1058.08, 892.05, 598.84, and 446.02 cm^−1^, were shifted to lower wave number like 3406.48, 2340.29, 1739.67, 1633.05, 1405.59, 1018.21, 932.92, 572.19, and 438.92 cm^−1^, respectively. The shifting absorptions peaks are associated with the impregnation of iron oxide onto biomass, MAFP.

**Figure 5:**
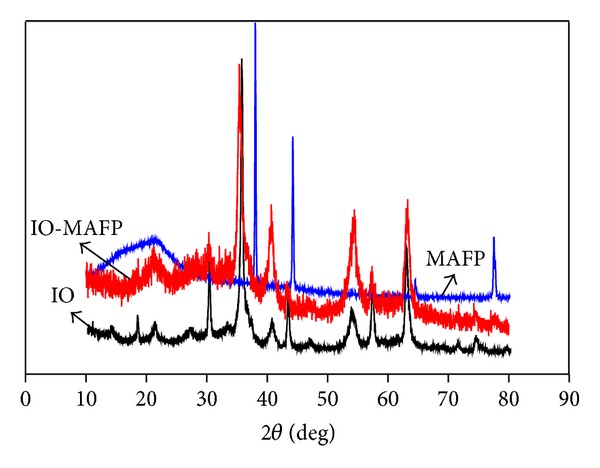
XRD spectrum of MAFP, Iron oxide, and IO-MAFP alone.

X-ray diffraction studies have been carried out on iron oxide and iron oxide impregnated *Morus Alba* L. fruit peel (IO-MAFP) (Figure 5). Bragg reflections in the X-ray diffraction patterns point out the crystalline structure of the end products. It is clear from the X-ray diffraction spectra that after iron oxide impregnation onto *Morus Alba* L. fruit peel presence of maghemite (*γ*-Fe_2_O_3_) was observed which indicates the impregnation of iron oxide onto MAFP, which in turn assist the adsorption of Co(II).

### 3.2. Optimization of Batch Biosorption

Biosorption of Co(II) onto the surface of the biomass, MAFP, and IO-MAFP is affected by several factors, such as biomass concentration, solution pH, initial metal ion concentration, time, and temperature.

#### 3.2.1. Effect of Biomass Dosage

**Figure 6:**
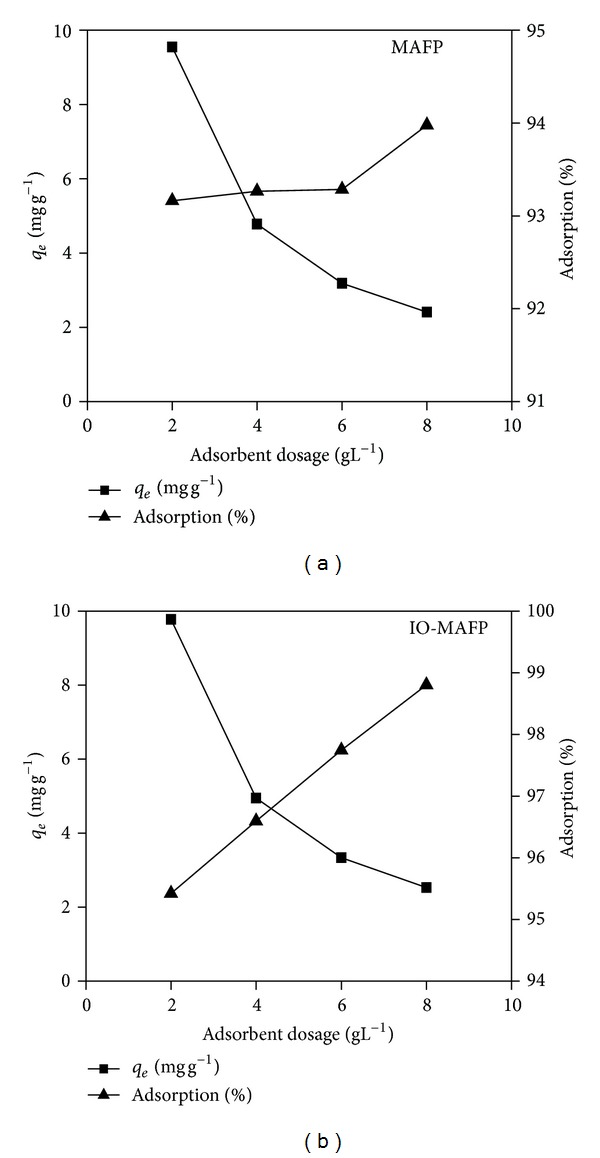
Biomasses, MAFP, and IO-MAFP dosage effect on sorption of Co(II).

The number of sites available for biosorption depends upon the amount of the sorbent. The effect of the sorbent concentration (2.0–8.0 g L^−1^) on the metal (20 mg L^−1^) removal efficiency was studied at pH 6.0 in a temperature (25 ± 2°C) controlled water bath for 60 min with 200 rpm and the results are shown in Figure 6. The percentage of metal ions uptake was found to be increased with the increasing concentration of the biosorbent but the amount of metal adsorbed for unit mass was decreased considerably. The increase in the removal percentage is due to the increase in active sites on the sorbent and thus making easier penetration of the metal ions to the sorption sites. But, decrease in unit sorption with increasing in the dose of both sorbents. It may be due to the formation of sorbent agglomerates reducing available surface area and blocking some of the sorption sites for unit mass of sorbent. However, the obtained results observed that the sorption capacity of Co(II) for unit mass of both sorbents was showing high at 2.0 g L^−1^ sorbent dosage. Even though sorption percentage of Co(II) is increasing, the sorption capacity for unit mass of both sorbents is decreasing considerably behind the 2.0 g L^−1^ dosage. Hence, we consider 2.0 g L^−1^ sorbent dosage a better one for further present investigations. 

#### 3.2.2. Effect of pH

**Figure 7:**
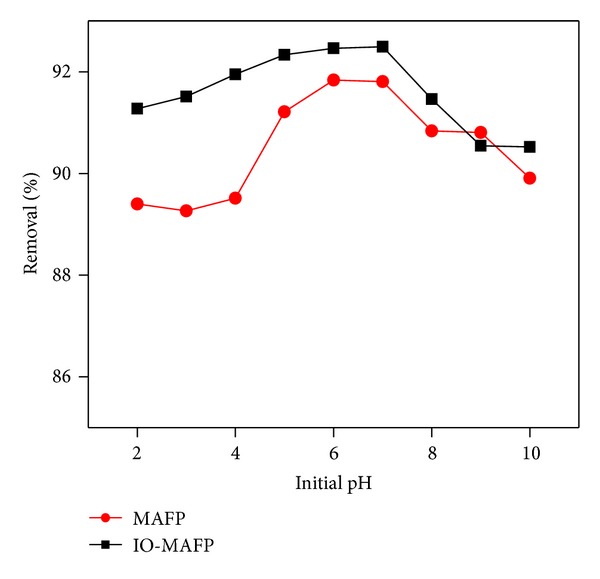
Evaluation of pH effect on sorption of Co(II) onto MAFP and IO-MAFP biomasses.

The pH of the solution is well-known characteristic that affects the surface charge of sorbents by the protonation of functional groups in the biomass, as well as the degree of ionization and chemistry of the metal ions. The optimum pH for Co(II) biosorption was investigated by adding 2.0 g L^−1^ MAFP or IO-MAFP biomasses to aqueous metal solution and was adjusted to various pH values (2.0 to 10.0) using 0.1 moL L^−1^ NaOH or 0.1 moL L^−1^ HCl. After adjusting the solution pH flasks were shaken for 60 min at 200 rpm and room temperature (25 ± 2°C). As shown in Figure 7, the metal uptake increased with the increase of pH in the range of 2.0 to 7.0 and after above pH 7.0 it was decreased to lower value for both sorbents. The plausible explanation for the lower sorption capacity observed at lower pH is due to the fact that the concentrations of protons and hydronium (H_3_O^+^) ions were higher and this competes for the binding of active sites on the surface of the sorbent with metal ions. Further with increasing pH there is a decrease in competition between the protons surrounded by the sorbent and metal ions. When the pH of the solution was increased from pH 2.0 to 7.0 the number of negatively charged sites increased resulting in increased sorption capacity of Co(II) [46]. Further the sorption capacity of Co(II) was decrease to lower value from pH 7.0 to 10.0. This decrease in sorption may be attributed to reduced solubility and precipitation of Co(II) [47]. Hence, pH 6.0 was chosen as optimum initial pH for further sorption studies. 

#### 3.2.3. Effect of Ionic Strength on Biosorption

Wastewaters from industries consist of various types of suspended and dissolved compounds apart from the metal ions. These impurities could be acids, alkalis, salts, or metal ions. The effect of ionic strength on cobalt biosorption was studied by changing NaCl concentration from 0.005 to 0.045 moL L^−1^ which is the level of salt in natural water at room temperature (25 ± 2°C) and 10 mg L^−1^ Co(II) initial concentration. The obtained results indicate that the increased concentration of ionic strength led to a slight decrease in the amount of adsorbed Co(II) this may be due to the competitive interaction of NaCl and Co(II) with surface active sites of biosorbent. However, the amount of adsorbed Co(II) was not significantly affected with ionic strength.

#### 3.2.4. Effect of Contact Time and Initial Metal Ion Concentration

**Figure 8:**
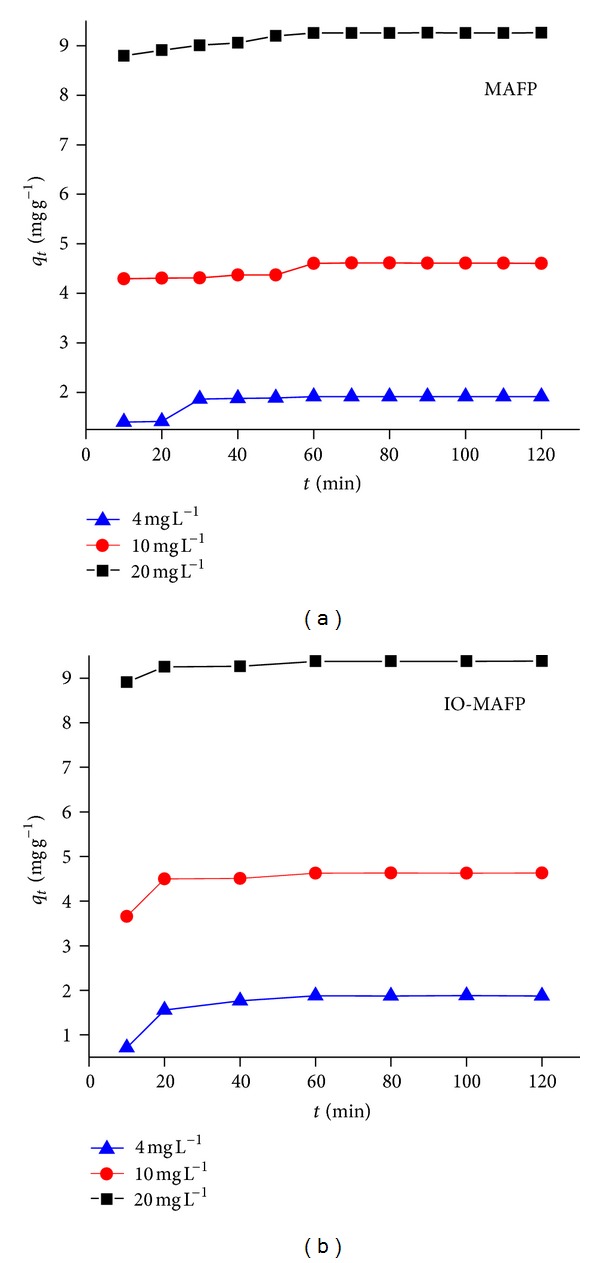
Effect of contact time and initial metal ion concentration on biosorption of Co(II) using MAFP and IO-MAFP biomasses.

The contact time was also evaluated as one of the most important factors affecting the biosorption efficiency.  Figure 8 shows the biosorption efficiency of metal ions (initial concentration, 4.0–20.0 mg L^−1^) by MAFP and IO-MAFP sorbents (2.0 g L^−1^) as a function of contact time (10 to 120 min) in a temperature controlled shaking water bath at 25 ± 2°C. It has been observed that metal ion sorption was rapid at initial stage (within the first 20 min); after this it was relatively slow until it reaches the equilibrium. The plausible reason is that a large number of vacant surface sites are available for adsorption at first, and after a lapse of time the remaining vacant surface sites are difficult to be occupied due to repulsive forces between the solute molecules on the solid and aqueous phases. Similar results were observed by some researcher with different sorbate-sorbent system [48–51]. Based on the results 60 min was fixed for further batch sorption experiments to assume that the equilibrium is achieved. As shown in Figure 8, with the increase in initial concentration of Co(II) from 4.0 to 20.0 mg L^−1^, the absolute sorption per unit mass of biosorbent increased. However, the percentage of Co(II) biosorption decreases with increasing initial concentration. It may be due to the fact that the available active sorption sites became fewer at higher initial concentration. It concluded that the Co(II) biosorption is concentration dependent.

### 3.3. Biosorption Kinetics

Two kinetic models including pseudo-first-order and pseudo-second-order models were used to fit the sorption data of Co(II) onto MAFP and IO-MAFP. The conformity between experimental data and the model predicted values was expressed by correlation coefficient (*R* ^2^) and checking the closeness of equilibrium sorption capacities (*q*_*e*_) obtained by model and experimental data at equilibrium.

The pseudo-first-order model [52] describes the rate of sorptionto be proportional to the number of sites unoccupied by the solutes. The expression of this model is as follows:

(4) $$\begin{array}{c} \log\left(q_{e}-q_{t}\;\right)=\log\left(q_{e}\;\right)-\left(\frac{K_{1}}{2.303}\right)t. \end{array}$$

**Figure 9:**
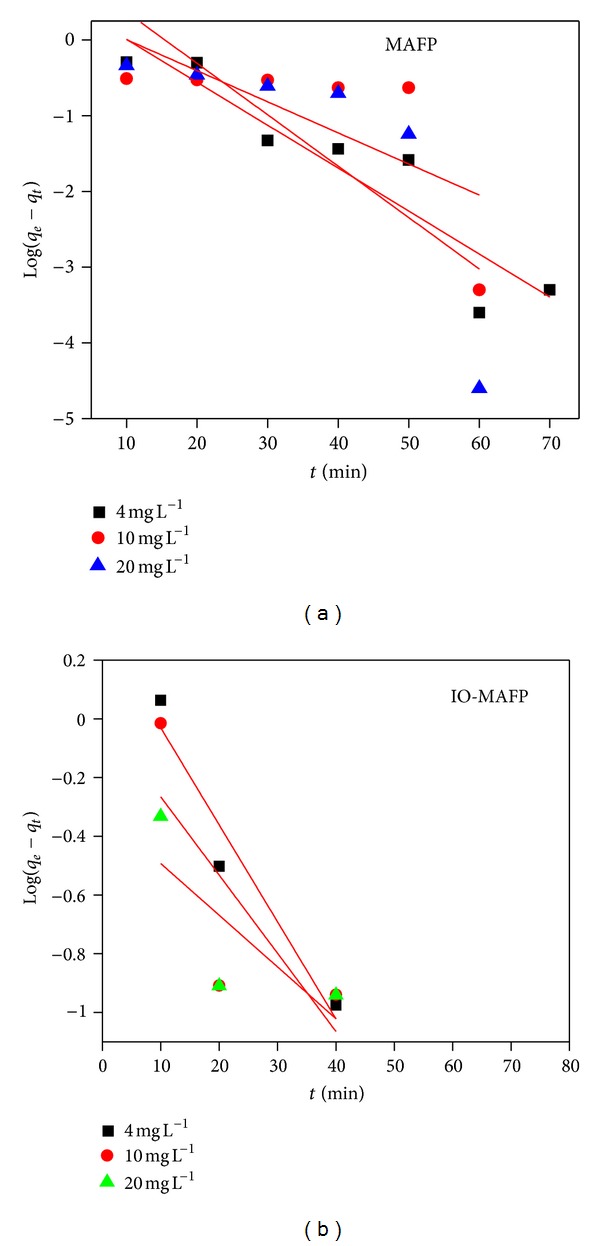
Pseudo-first order for Co(II) biosorption onto MAFP and IO-MAFP biomasses.

**Figure 10:**
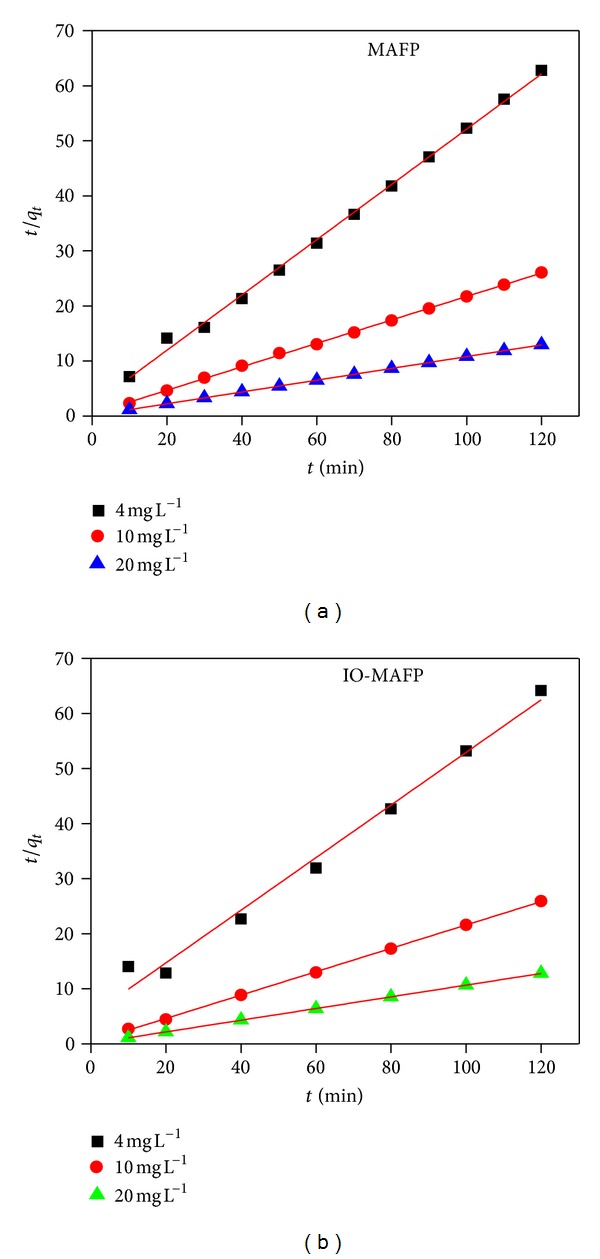
Pseudo-second order for Co(II) biosorption onto MAFP and IO-MAFP biomasses.

Ho and McKay [53] noticed that the use of the Lagergren model for prediction of the biosorption kinetics is not suitable for the entire sorption period. The pseudo-first-order model works effectively only in the region where biosorption process occurs rapidly. The pseudo-second-order kinetic model is expressed as [53]

(5) $$\begin{array}{c} \frac{t}{q_{t}}=\frac{1}{K_{2}\;q_{e}^{2}}\;+\left(\;\frac{1}{q_{e}}\right)t, \end{array}$$

where *q*_*t*_ and *q*_*e*_ are the metal ion concentrations (mg g^−1^) at time (*t*) and the equilibrium (mg g^−1^), respectively, and *K*_1_ (min^−1^) and *K*_2_ (g mg^−1^ min^−1^) are the rate constant of pseudo-first-order and second-order kinetics, respectively. The values constants *K*_1_ and  *K*_2_  were calculated from slope of the model curve (Figures 9 and 10), equilibrium sorption capacities (*q*_*e*_) obtained by model, and experimental data at equilibrium and correlation coefficient (*R* ^2^) for both sorbents was summarized in Table 2. The correlation coefficient and the large difference of equilibrium sorption capacities (*q*_*e*_) obtained by model and experimental data at equilibrium for biosorption of Co(II) by MAFP and IO-MAFP indicate that the pseudo-first-order kinetics was not fitted well to biosorption kinetic data. 

It was observed that pseudo-second-order kinetics model (Figure 10) showed best fit with high correlation coefficient values range from 0.985 to 0.999. Further low difference in equilibrium sorption capacity *q*_*e*_ (mg/g) values (Table 2) obtained by experimental data and model at equilibrium which indicates that the sorption of Co(II) onto MAFP and IO-MAFP follows the pseudo-second-order kinetics. From comparison of the two models, it can be concluded that the sorption of Co(II) onto MAFP and IO-MAFP follows the rate limiting pseudo-second-order kinetics. 

### 3.4. Biosorption Isotherms

A biosorption isotherm characterized certain values, which express the surface properties and affinity of the biosorbent and can also be used to compare the biosorption capacities of the biosorbent for different pollutants [54, 55]. Sorption equilibrium data can be described by a number of isotherm models available in the literature. In this study, Langmuir, Freundlich, and Temkin isotherm models were selected to fit experimental data at three different temperature values 298, 313, and 328 K. 

**Figure 11:**
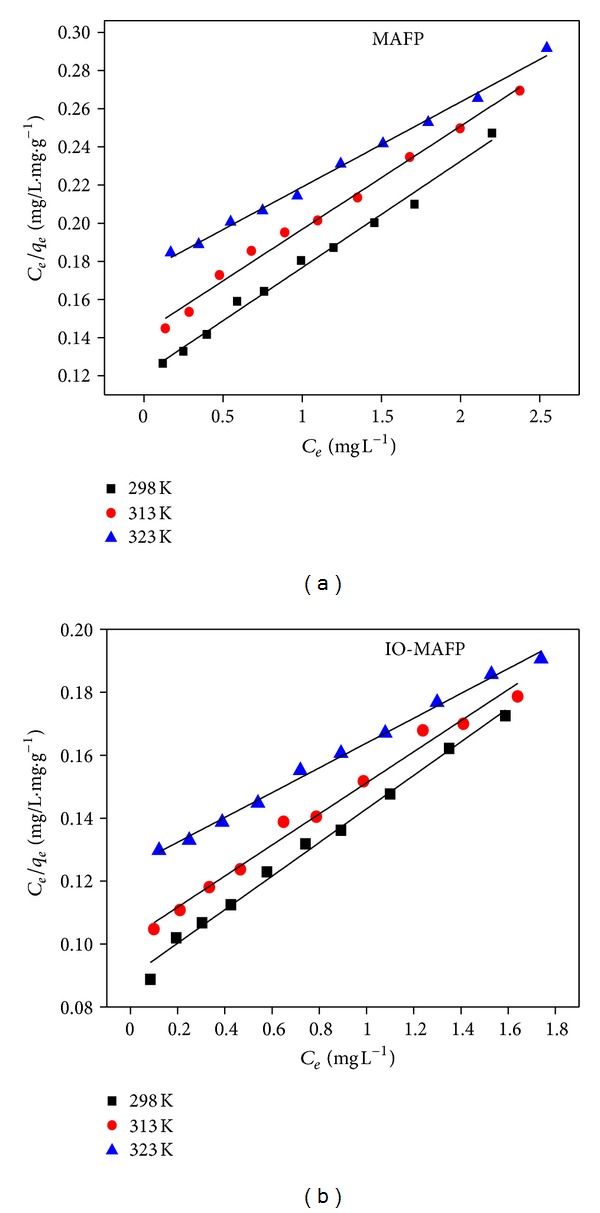
Langmuir isotherms of Co(II) sorption onto MAFP and IO-MAFP biomasses.

Langmuir [56] model supposes that the sorption process takes place at a specific sorption surface and was expressed as

(6) $$\begin{array}{c} \frac{C_{e}}{q_{e}}=\frac{1}{q_{\max}\left(1/K_{L}\;\right)}+\frac{1}{q_{\max}\left(C_{e}\;\right)}, \end{array}$$

where *q*_max⁡_ and *K*_*L*_ indicate the maximum adsorption capacity of adsorbent and Langmuir equilibrium constant, respectively. These were obtained from the slope (1/*q*_max⁡_) and intercept (1/*q*_max⁡_ *K*_*L*_) of the linear fit of the plot between *C*_*e*_/*q*_*e*_ versus *C*_*e*_ (Figure 11) at 298, 323, and 328 K and were summarized in Figure 11 and Table 3. The correlation coefficients (*R* ^2^) of the curves were in the range from 0.988 to 0.996 for Co(II) biosorption at 298, 323, and 328 K system temperature for the both biosorbents. The obtained results indicated that the biosorption of Co(II) onto MAFP and IO-MAFP follows Langmuir isotherm model. The obtained results also concluded that the maximum adsorption capacity was increased with increasing system temperature in the range from 298 to 328 K.

The essential characteristics of the Langmuir isotherm can be also expressed in terms of a dimensionless constant separation factor *R*_*L*_ and is expressed as follows [57]:

(7) $$\begin{array}{c} R_{L}\;=\;\frac{1}{\left(1+K_{L}\;C_{o}\right)}, \end{array}$$

where *C*_*o*_ is the highest initial concentration of adsorbate (mg L^−1^)and *K*_*L*_ (L mg^−1^) is the Langmuir constant. The *R*_*L*_ values indicate the shape of the isotherm to be either favorable (0 < *R*_*L*_ < 1), linear (*R*_*L*_ = 1), unfavorable (*R*_*L*_ > 1), or irreversible (*R*_*L*_ = 0) [57, 58]. The *R*_*L*_ values (Table 3) in the present investigations were found to be between 0.013 and 0.029 at 298, 313, and 323 K for the both the sorbent. These values were in the range of 0 < *R*_*L*_ < 1 indicating that the sorption of Co(II) on MAFP and IO-MAFP biomasses is favorable.

**Figure 12:**
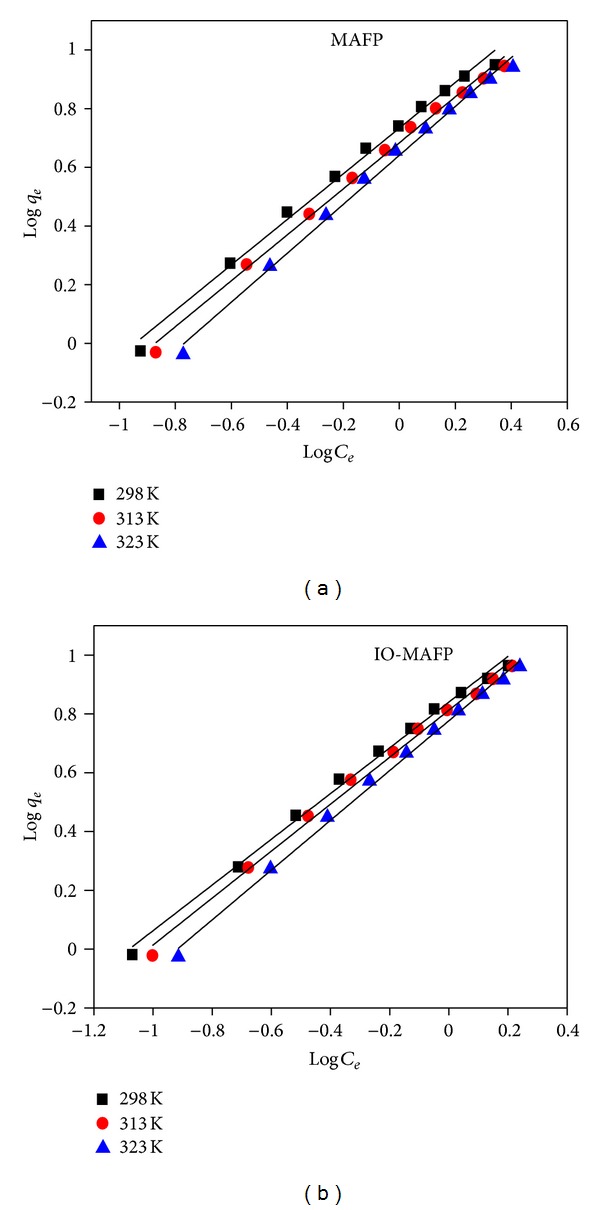
Freundlich isotherm of Co(II) sorption oto MAFP and IO-MAFP biomasses.

The Freundlich [59] isotherms are an empirical expression that takes into account the heterogeneity of the surface and multilayer adsorption to the binding sites located on the surface of the sorbent. The Freundlich isotherm model equation is represented as follows:

(8) $$\begin{array}{c} \ln q_{e}=\ln K_{F}+\frac{1}{n\;\ln C_{e}}, \end{array}$$

where *K*_*F*_ is the biosorption equilibrium constant and *n* is an empirical constant which indicates the biosorption intensity. These constants were obtained from the slope (1/*n*) and intercept (ln⁡*K*_*F*_) of the plot between log *C*_*e*_ and log *q*_*e*_ (Figure 12) and were summarized in Table 3. The correlation coefficients (*R* ^2^) of the curves were in the range from 0.993 to 0.996 for sorption of Co(II) onto MAFP and IO-MAFP sorbents at 298, 323, and 328 K system temperature which indicates that the Freundlich isotherm model was well fitted. It also concluded that the *K*_*F*_ decreases while 1/*n* increases as increasing system temperature.

Temkin and Pyzhev [60], considered the effects of indirect sorbate/sorbate interactions on sorption isotherms. This isotherm model can be expressed in linear form as

(9) $$\begin{array}{c} q_{e}=B\;\ln K_{T}+B\;\ln C_{e}, \end{array}$$

where  *B* = (*RT*/*b*), which is obtained from the slope of plot *q*_*e*_ versus ln⁡*C*_*e*_. The intercept of curve indicates *B*  ln⁡*K*_*T*_. The Temkin isotherm constants *B* · *KT* is related to maximum binding energy and *B* is corresponding to heat adsorption. The heat of adsorption of all the molecules in the layer would decrease linearly with coverage due to sorbate molecules interactions. It was not well fitted to the sorption data of Co(II) onto both sorbents was predicted by the *R* ^2^ values of this isotherm (Table 3).

From Table 3, it was observed that both the Langmuir and Freundlich isotherm models were yielded best fit as indicated by the highest *R* ^2^ values at system temperature compared to Temkin adsorption isotherm model. The order of isotherms models fitted to the Co(II) sorption onto MAFP and IO-MAFP was as follows: Freundlich ≥ Langmuir > Temkin. Best fitting of the equilibrium data with Langmuir and Freundlich isotherms suggests that biosorbent surface contains both homogeneous and heterogeneously distributed active sites.

### 3.5. Comparison of Present Biosorption Capacity with Other Biosorbents

Biosorption capacities of various biosorbents towards Co(II) removal reported in literature were compared with the MAFP and IO-MAFP biosorbents and the results are summarized in Table 4. From the results it was found that the maximum sorption capacity of MAFP and IO-MAFP is found to have a relatively large sorption capacity of 17.953 and 18.762 mg/g at 298 ± 2 K for both sorbents, respectively. This indicates that these biomasses could be considered promising materials for the removal of Co(II) ions from aqueous solutions. From the results it was clear that the present biomasses are well suitable to be used as sorbents for sorption of Co(II) ions. It was also concluded that the impregnation of iron oxide onto MAFP enhances its sorption capacity towards higher level. It was clear from the results that MAFP and IO-MAFP biomasses appear to be economic as well as efficient sorbents for the Co(II) removal from aqueous solutions.

### 3.6. Biosorption Mechanism

The FT-IR spectrum of Co(II) loaded biomasses was shown in Figure 4. It is observed that the absorption peaks of –NH_2_, –CONH_2_, aromatic and aliphatic –COOH groups, –C=O, and –C–X (X= S^2−^, halides, O^2−^) in MAFP and IO-MAFP were shifted to higher wave number by the loading of Co(II) into MAFP and IO-MAFP. This shifting of absorption peaks was concluded that the involvement of Co(II) with –NH_2_, –CONH_2_, aromatic and aliphatic –COOH groups, and –C=O and –C–X (X= S^2−^, halides, O^2−^) groups by the adsorption phenomena on the both biomasses.

Further SEM-EDX analysis was carried out and the SEM morphology of biomass and metal loaded biomasses was shown in Figure 2. The surface morphology of Co(II) loaded biomasses (Figure 2) was different than original biomasses, where shiny and bright white surface morphology was observed, indicating the Co(II) metal ions adsorbed on the surface of biomasses. The presence of sulfur in MAFP is advantageous, because sulfur groups, which are soft bases, have chemical affinity towards cobalt. Thus presence of sulfur in MAFP qualifies it as a potential sorbent. From the overall results it was found that chemisorption plays an important role in the removal of Co(II) from aqueous solution by both sorbents. However, the impregnation of iron oxide into MAFP enhances the sorption capacity of MAFP towards higher level with good desorption nature.

### 3.7. Thermodynamic Studies of Biosorption

**Figure 13:**
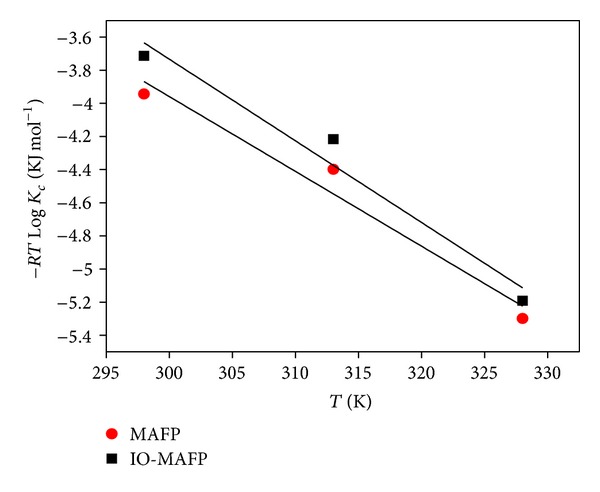
Biosoption thermodynamics of Co(II) onto MAFP and IO-MAFP biomasses.

The thermodynamics studies of adsorption are an important parameter to describe the interactions between sorbent and sorbate as well as energy changes during sorption mechanism. In the environmental engineering practices, the change in entropy and energy should be measured in order to determine the processes that occur spontaneously. Thermodynamic parameters, the change in Gibb's free energy (Δ*G* ^*o*^), enthalpy (Δ*H* ^*o*^), and entropy (Δ*S* ^*o*^) of the present system were determined using the following equation:

(10) $$\begin{array}{c} \Delta G^{o}=-RT\;\ln K_{c},\\ \Delta G^{o}=\Delta H^{o}-T\;\Delta S^{o}, \end{array}$$

where *R* is the universal gas constant (8.314 × 10^−3^ kJ moL K^−1^), *T* is the temperature of the system in K, *K*_*c*_ is the equilibrium constant which was the product of maximum amount adsorbed by sorbent (*q*_max⁡_) and equilibrium constant (*K*_*L*_) at Langmuir equilibrium. The values of Δ*H* ^*o*^ and Δ*S* ^*o*^ were calculated from the intercept and slope of the linear curve between Δ*G* ^*o*^ verses *T* (Figure 13) and were reported in Table 5. 

From the obtained results (Table 5) the negative values of Δ*G* ^*o*^ indicate the spontaneous nature of biosorption mechanism on both sorbents. The positive values of Δ*H* ^*o*^ and Δ*S* ^*o*^ indicated the randomness of the sorbate-sorbent interaction with endothermic nature of sorption mechanism of Co(II) on both sorbents in the temperature range of 298–328 K [61, 62]. One plausible explanation of endothermicity of the enthalpy of sorption is the well-known fact that metal ions are well solvated in water. There needs to extend some energy to this ion dehydration from its hydration sheath in aqueous solution in order to get sorption of Co(II) on the sorbent. This dehydration process energy assumes that exceeds the exothermicity of the ion attaching to the surface. The implicit assumption is that after sorption, the environment of the metal ions is less aqueous than that in the solution state. The removal of water from ions is essentially an endothermic process exceeds that of the enthalpy of sorption to considerable extent [63, 64]. 

### 3.8. Desorption Studies of Biosorption

Sodium salt of EDTA, hydrochloric acid, nitric acid, and sodium carbonate solutions were used for the desorption studies of Co(II) from biomass. As the concentration of desorbing solutions increases, the desorption amount of Co(II) ions from loaded biomasses increases. The obtained results were reported in Table 6 indicating that more than 90% of Co(II) ions were able to be desorbed from both biomasses using 0.03 moL L^−1^ hydrochloric acid, nitric acid, and EDTA solutions. Especially, > 98% recovery of Co(II) was achieved with 0.03 moL L^−1^ EDTA and nitric acid from the both sorbents, MAFP, and IO-MAFP. However, 99.15% Co(II) desorption was achieved with EDTA from IO-MAFP. The obtained results concluded that the tested sorbents could be reused without significant losses in its initial sorption capacity. It might help to elucidate the sorption and desorption behavior of Co(II) in aqueous solutions for recovery and recycling of sorbent at particular treatment of effluents.

## 4. Conclusions

MAFP and IO-MAFP obtained from fruit peel biomass were an ecofriendly potential biosorbent for Co(II) removal. Biosorption is affected by various parameters, such as biomass concentration, pH, and temperature. The kinetic studies revealed that the biosorption process followed the pseudo-second-order kinetic model for the both sorbents. Freundlich, Langmuir, and Temkin adsorption isotherm models applied to the biosorption data of Co(II) for evaluation of sorption efficiency of the biosorbents; these were in the order: Freundlich ≥ Langmuir > Temkin. Best fitting of the equilibrium data with Langmuir and Freundlich isotherms suggest that biosorbent surface contains both homogeneous and heterogeneously distributed active sites. The maximum biosorption capacity of Co(II) was 17.953 and 18.762 mg/g at 298 ± 2 K and an optimum pH 6.0. The biosorption capacities of present MAFP and IO-MAFP were near or more than the reported results of various biosorbents indicating that the present biosorbents were considered to be promising and potential materials for Co(II) removal. The impregnation of iron oxide onto MAFP enhances its sorption capacity towards higher level. The negative values of Δ*G* ^*o*^ and the positive values of Δ*H* ^*o*^ and Δ*S* ^*o*^ indicate the spontaneous and randomness of the sorbate-sorbent interaction with endothermic nature of sorption mechanism of Co(II) on both sorbents in the range 298–328 K. This suggests that these biosorbents can be successfully employed for the regular adsorption/biosorption of metal ions in the large scale from metallurgical industries.

## Figures and Tables

**Table 1:** Physical characteristics of MAFP and IO-MAFP biosorbent.

Physical characteristics	Values	
	MAFP	IO-MAFP
Bulk density	0.35~0.42 g mL^−1^	0.40~0.55 g mL^−1^
Moisture content	1.23%	1.30%
Ash content	12.53%	11.42%
Surface area	16.29 m^2^ g^−1^	335.25 m^2^ g^−1^
Pore volume	0.093 cm^3^ g^−1^	12.27 cm^3^ g^−1^
Pore mean diameter	15.27 × 10^−8^ cm	55.5 × 10^−8^ cm

**Table 2:** Kinetics parameters of Co(II) biosorption with MAFP and IO-MAFP.

Type of Adsorbent	Initial metal ion concentration, mg L^−1^	*q*_*e*,exp._, mg g^−1^	Pseudo-first-order			Pseudo-second-order			
			*q*_*e*_, mg g^−1^	*K*_1_, min^−1^	*R* ^2^	*q*_*e*_, mg g^−1^	*K*_2_, g mg^−1^ min^−1^	*h*, mg g^−1^ min^−1^	*R* ^2^
MAFP	4	1.876	3.778	0.131	0.846	1.993	0.129	0.516	0.998
	10	4.621	2.606	0.095	0.340	4.697	0.106	2.329	0.999
	20	9.001	11.189	0.156	0.504	9.337	0.129	11.261	0.999

IO-MAFP	4	1.903	1.992	0.0761	0.885	2.094	0.044	0.194	0.985
	10	4.711	1.005	0.0613	0.201	4.714	0.119	2.666	0.999
	20	9.280	0.482	0.0405	0.232	9.426	0.129	19.172	0.999

**Table 3:** Isotherm parameters of Co(II) with MAFP alone and IO-MAFP biosorbent.

Name of Adsorbent	Temperature, K	Langmuir isotherm parameters				Freundlich isotherm parameters			Temkin Isotherm parameters		
		*q*_*m*_, mg g^−1^	*K*_*L*_, L mg^−1^	*R* ^ 2^	*R*_*L*_	*K*_*F*_, mg g^−1^ (L mg^−1^)^1/^ *n *	1*/n *	*R* ^ 2^	*K*_*T*_, L mg^−1^	*B *	*R* ^ 2^
MAFP	298	17.953	2.174	0.992	0.023	5.42	0.777	0.993	8.138	6.567	0.948
	313	18.519	2.645	0.991	0.019	4.831	0.784	0.996	7.063	6.513	0.941
	328	22.422	3.910	0.996	0.013	4.375	0.834	0.995	5.646	6.864	0.945

IO-MAFP	298	18.762	1.681	0.992	0.029	6.917	0.777	0.996	10.912	6.702	0.941
	313	20.243	2.061	0.988	0.024	6.491	0.798	0.996	9.528	6.845	0.940
	328	25.381	3.159	0.996	0.016	5.979	0.846	0.995	7.721	7.184	0.937

**Table 4:** Comparison of maximum biosorption capacities (*q*_max⁡_, mg/g) of Co(II) with various biosorbent at 25°C.

Biosorbent	Biosorption capacity, mg g^−1^
*Ficus religiosa *(peepal) [22]	3.60
*Hypogymnia physodes* (Foliose lichen) [23]	9.90
*Evernia prunastri *(fruticose lichen) [24]	5.72
*R. arrhizus* (fungi) [25]	2.90
*Saccharomyces cerevisiae* [25]	5.80
*Rhytidiadelphus squarrosus *(moss) [26]	7.25
MAFP*	17.953
IO-MAFP*	18.762

*Present study.

**Table 5:** Thermodynamic parameters of Co(II) biosorption on MAFP and IO-MAFP.

Name of adsorbent	Temperature, K	log⁡*K*_*C*_	Δ*G*°, KJ mol^−1^	Δ*H*°, KJ mol^−1^	Δ*S*°, KJ mol^−1^ K
MAFP	298	1.591	−3.943	9.595	0.045
	313	1.690	−4.398		
	328	1.943	−5.298		

IO-MAFP	298	1.499	−3.714	11.056	0.049
	313	1.620	−4.217		
	328	1.904	−5.193		

**Table 6:** Desorption studies of Co(II) from MAFP and IO-MAFP biomasses.

Desorbing solution	Concentration of desorbing solution mol L^−1^	Desorption of metal ion (%)	
		MAFP	IO-MAFP
EDTA	0.001	56.45	60.54
	0.005	76.25	80.32
	0.01	87.24	90.31
	0.02	90.25	93.50
	0.03	98.06	99.15

HCl	0.001	22.56	25.43
	0.005	36.57	35.07
	0.01	85.28	87.76
	0.02	89.96	88.95
	0.03	92.15	91.23

HNO_3_	0.001	33.46	32.65
	0.005	49.98	53.46
	0.01	78.26	81.23
	0.02	90.26	92.45
	0.03	98.24	98.76
